# Direct *In Vivo* Evidence for Tumor Propagation by Glioblastoma Cancer Stem Cells

**DOI:** 10.1371/journal.pone.0024807

**Published:** 2011-09-22

**Authors:** Justin D. Lathia, Joseph Gallagher, Jay T. Myers, Meizhang Li, Amit Vasanji, Roger E. McLendon, Anita B. Hjelmeland, Alex Y. Huang, Jeremy N. Rich

**Affiliations:** 1 Department of Stem Cell Biology and Regenerative Medicine, Lerner Research Institute, Cleveland Clinic, Cleveland, Ohio, United States of America; 2 Department of Pediatrics, Case Western Reserve University School of Medicine, Cleveland, Ohio, United States of America; 3 Image Processing Core, Lerner Research Institute, Cleveland Clinic, Cleveland, Ohio, United States of America; 4 Department of Pathology, Duke University Medical Center, Durham, North Carolina, United States of America; 5 Molecular Medicine Program, Cleveland Clinic Lerner College of Medicine at Case Western Reserve University School of Medicine, Cleveland, Ohio, United States of America; University of Chicago, United States of America

## Abstract

High-grade gliomas (World Health Organization grade III anaplastic astrocytoma and grade IV glioblastoma multiforme), the most prevalent primary malignant brain tumors, display a cellular hierarchy with self-renewing, tumorigenic cancer stem cells (CSCs) at the apex. While the CSC hypothesis has been an attractive model to describe many aspects of tumor behavior, it remains controversial due to unresolved issues including the use of *ex vivo* analyses with differential growth conditions. A CSC population has been confirmed in malignant gliomas by preferential tumor formation from cells directly isolated from patient biopsy specimens. However, direct comparison of multiple tumor cell populations with analysis of the resulting phenotypes of each population within a representative tumor environment has not been clearly described. To directly test the relative tumorigenic potential of CSCs and non-stem tumor cells in the same microenvironment, we interrogated matched tumor populations purified from a primary human tumor transplanted into a xenograft mouse model and monitored competitive *in vivo* tumor growth studies using serial *in vivo* intravital microscopy. While CSCs were a small minority of the initial transplanted cancer cell population, the CSCs, not the non-stem tumor cells, drove tumor formation and yielded tumors displaying a cellular hierarchy. In the resulting tumors, a fraction of the initial transplanted CSCs maintained expression of stem cell and proliferation markers, which were significantly higher compared to the non-stem tumor cell population and demonstrated that CSCs generated cellular heterogeneity within the tumor. These head-to-head comparisons between matched CSCs and non-stem tumor cells provide the first functional evidence using live imaging that in the same microenvironment, CSCs more than non-stem tumor cells are responsible for tumor propagation, confirming the functional definition of a CSC.

## Introduction

Human tumors commonly display a heterogeneity within their neoplastic compartment that may be derived from a combination of stochastic genetic copy number alterations and an epigenetic hierarchy that co-evolve over time [1]. Integrating the concept that tumors may contain a stem cell-like population responsible for their maintenance and propagation may be informative for both the cancer and stem cell fields [2]. The CSC hypothesis may provide insights into therapeutic resistance and tumor recurrence and underscore the complexity of cancer. The excitement surrounding the CSC hypothesis is tempered by controversy with regard to appropriate experimental model systems to functionally define CSCs, CSC frequency, and universally informative immunophenotypes [3]. Normal and neoplastic stem cells are currently defined by functional assays of self-renewal and differentiation, with the most accurate assay to date for CSCs being tumor propagation. Xenotransplantation models have confirmed the enhanced tumor formation capacity of the CSC-enriched fractions in a variety of human tumors and have been used to estimate the frequency of tumor propagating cells [4], which is quite high for some malignancies [3]. As the niche in which both normal and neoplastic stem cells reside instructs self-renewal and maintenance, live animal in vivo imaging techniques have been applied to some stem cell populations – notably hematopoietic and leukemic stem cells – to determine growth patterns in the native microenvironment [5], [6], [7], but the application to solid tissues has been limited. Solid tumor CSCs have been characterized in ex vivo assays or as segregated populations, which have been informative in determining differentially regulated pathways but have prevented the direct analysis of tumor propagation potential between different tumor cell fractions. To evaluate the potential of CSCs in direct comparison to non-stem tumor cells in a representative microenvironment, we differentially labeled GBM cell fractions derived from a human tumor and monitored tumor behavior in a xenotransplantation model over time using intravital microscopy. Despite small numbers of CSCs at transplantation, tumor propagation was driven by CSCs and their descendants, demonstrating the ability for CSCs, but not non-stem tumor cells, to drive tumor formation and propagate cellular heterogeneity.

## Materials and Methods

### Transplantation of glioma cells

Human glioma cells were derived with written informed consent and under approved IRB protocols from Cleveland Clinic (Protocol 2559) and Duke University (Protocol 7409). Glioma cells were transiently passaged as xenografts in nude mice under approved Cleveland Clinic IACUC protocol ARC 8699 and in vivo imaging was performed under Case Western Reserve University Protocol 2009-0109). For initial CSC tumor formation studies, the tumor specimen used (T4302) was a newly diagnosed grade III anaplastic astrocytoma in a 40 year old male which was surgically removed at Duke University. At time of removal, the specimen was characterized to have EGFR polysomy (EGFRvIII negative), MGMT negative, intact PTEN, and polysomy of chromosomes 7 and 10. For cell mixing studies (i.e. competition assay), the tumor specimen used (T4121) was a recurrent glioblastoma multiforme (GBM) diagnosed in a 26 year old male which was surgically removed at Duke University. At time of removal, the specimen was characterized to have amplified EGFR (but EGFRvIII negative), MGMT positive, PTEN loss, loss of chromosome 9p21, and polysomy of chromosomes 1p36, 1p32, and 19q13. For experimental studies, tumor cells were removed from xenografts and CSCs were enriched based on CD133 expression by flow cytometry (using a CD133/2-APC antibody, Miltenyi) then functionally assayed for self renewal, multi-lineage differentiation, stem cell marker expression and tumor propagation as previously described [8]. Putative CSCs from GBM specimen T4302 were transduced with a lentivirus to express green fluorescent protein (GFP). For tumor specimen T4121, putative CSCs were labeled with yellow fluorescent protein (YFP) or and non-stem tumor cells were labeled with a cyan fluorescent protein (CFP, tumor specimen T4121) by lentivirus (Sigma). Labeled CSCs and non-stem tumor cells (derived from T4121) were mixed at a 10%:90% [or 1∶9] (CSC:non-stem tumor cell) ratio and transplanted into the cortex of a nude mouse at a depth of 1.5 - 2 mm. Cranial windows were installed as previously described [9]. Imaging studies utilized 25,000 transplanted cells. All surgical procedures were done under an approved Cleveland Clinic IACUC protocol.

### Multiphoton imaging

Intravital microscopy was performed using multiphoton imaging as previously described [10] using a Leica SP5 imaging system with a 16W femto-second laser tuned to 840–860 nm and focused through a 20X water immersion lens (numeric aperture of 1.0). Prior to imaging, mice were anesthetized and intravenously injected with high-molecular weight fluorescent dextran (>150 kD) to highlight vasculature. Images were acquired over a 200 µm range in 2 µm z-stacks. Maximum intensity projections, representing 200 µm in depth, were uniformly adjusted in Photoshop (Adobe) prior to display. For three-dimensional reconstructions, data was imported into Imaris software (BitPlane) for surface rendering and volume was quantified for each cell population.

### Immunostaining and statistical analysis

Frozen sections were cut using a cryostat (Leica) and immunostaining was done as previously described [11] with antibodies against Tra1-85 (R&D Systems, 1∶500), Sox 2 (R&D Systems, 1∶200), phosphorylated Histone H3 (PH3, Millipore, 1∶500), and CD31 (Dako, 1∶200). Nuclei were counterstained with Draq5 (Biostatus limited, 1∶5000). Images were acquired using a Leica SP5 confocal microscope as previously described [11]. Analysis of the entire tumor was done for 3 anatomical depths in 3 representative mice, totaling <400,000 cells. Immunophenotyping of the transplanted cell populations was confirmed by flow cytometry using CD133 expression (CD133/2-APC antibody, Miltenyi) of each cell population as previously described [8]. Immunostaining on each cell population in vitro was done using antibodies described above and quantified based on a minimum of 150 cells from 10 fields. Statistical significance was calculated using one-way ANOVA.

### Automated image analysis

Large field-of-view (FOV) images of tumor cross-sections were acquired using a Leica DM4000, a 40X objective, a Q-Imaging CCD, a Prior 8-slide motorized stage, Image-Pro 6.2, and YFP, CFP, CY5 (DRAQ5 labeled nuclei), and Texas Red (Sox2, Tra-1-85, or PH3) filter cubes. Large FOV images (∼500 MB/channel) were imported into Image-Pro for analysis using customized macros. For each cross-section, the Draq5 channel was imported and spectrally enhanced to equalize the appearance of each nucleus. Nuclei were then segmented, morphologically “dilated” to extend their boundaries into the cytoplasm, and “watershed” filtered for cell separation. A region of interest (ROI) was drawn around the bulk tumor and CFP, YFP, and Texas Red channels were loaded in succession. Each channel was spectrally filtered and “multiplied” by the cell mask described above. Segmentation via morphometric and intensity profiles specific for each marker/stain followed by image logic operations provided YFP, CFP, and antibody positive cell counts. For immunophenotyping of the transplanted population, the resulting binary nuclear mask was “multiplied” with the corresponding PH3/Sox2 channel. Positivity was automatically assigned based on mean nuclear intensity of PH3 or Sox2 above a predefined threshold.

## Results

Malignant gliomas are solid cancers for which cellular hierarchies have been reproducibly defined, permitting the interrogation of cells that can be prospectively enriched for CSC characteristics, including self renewal, differentiation potential, stem cell marker expression, and in vivo tumor propagation. To address differential in vivo tumor propagation potential, we utilized models in which we had previously demonstrated the capacity to functionally enrich or deplete for CSC characteristics in ex vivo assays using fluorescent cell surface marker labeling [8], [11]. While there is controversy surrounding CD133 as a CSC marker, many proposed glioma CSC markers (including L1CAM [12], A2B5 [13], and integrin alpha 6 [11]) overlap with CD133. We find that CD133 enriches for CSCs in the GBM specimens used for the study and segregates for tumorsphere formation efficiency as compared to integrin alpha 6 (data not shown). Additionally, CD133 fractions efficiently propagate secondary tumors and CD133 enrichment of CSCs from these tumors has recently been used to validate a CSC-specific non-receptor tyrosine kinase and nitric oxide synthase isoform [14], [15]. CD133 expression was assessed after labeling by flow cytometry with 11% of YFP cells (CSC derived) and 1% of CFP cells (non-stem derived) being CD133 positive (data not shown). The viral labeling procedure did not alter growth properties (data not shown).

### *In vivo* observation of cancer stem cell growth into a tumor

**Figure 1:**
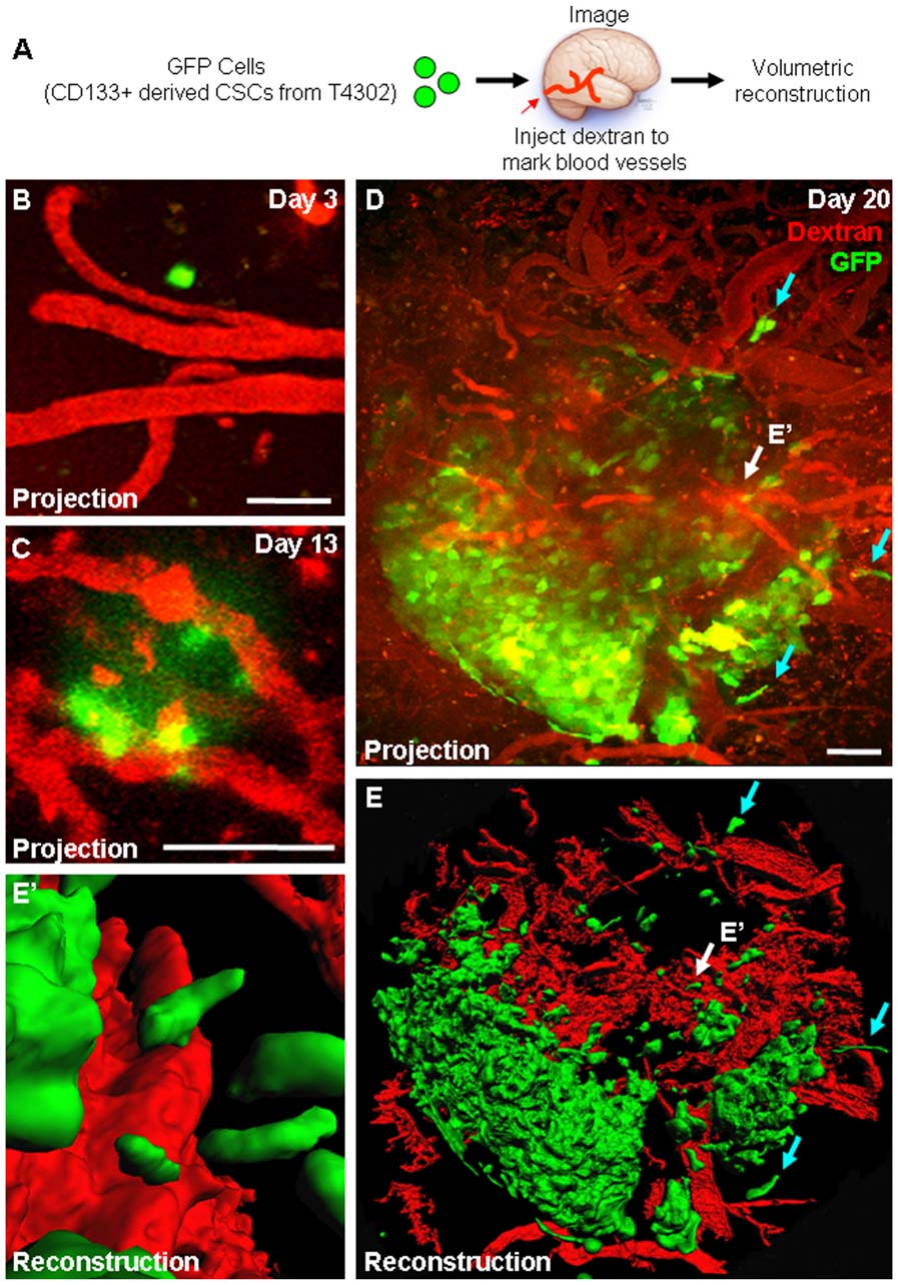
Multiphoton microscopy reveals tumor propagation from cancer stem cells. Tumor formation in a xenotransplantation model was observed from GFP-labeled CSCs over time as shown in experimental design schematic (**A**). Projection micrographs (**B**-**D**) demonstrate tumor formation over time and three-dimensional reconstructions depicted in micrographs (**E, E’**) revealed tumor cells were closely associated with blood vessels (**E’**, shown with white arrows in **D, E**) and in peripheral areas (**D**, **E**, shown in blue arrows). Fluorescent dextran (shown in red) was injected into the circulation to illuminate blood vessels prior to imaging. Scale bar represents 50 µm.

To date, the growth of solid tumor CSCs has not been evaluated at single cell resolution over a temporal time course in vivo. To observe the potential for tumor propagation by CSCs alone, we used intravital microscopy in an orthotopic xenotransplantation mouse model to identify and trace the growth of small numbers of CSCs into a tumor over time (Fig. 1A). Early in the observation period when limited amounts of CSCs were present (T4302, days 3 to 13), CSCs were associated with blood vessels in the perivascular niche (Fig. 1B,C) as described by Gilbertson and co-workers [16] and grew in proximity with blood vessels (Fig. 1D,E). Over time (from day 13 to 20), a tumor nodule rapidly formed and tumor cells were observed to infiltrate the peripheral regions (blue arrows), a common histological hallmark of malignant gliomas. The results provide the first tumor growth temporal time course of CSCs by live imaging and confirm the tumorigenic potential of the transplanted CSCs.

### Cancer stem cells outgrow non-stem tumor cells *in vivo*

**Figure 2:**
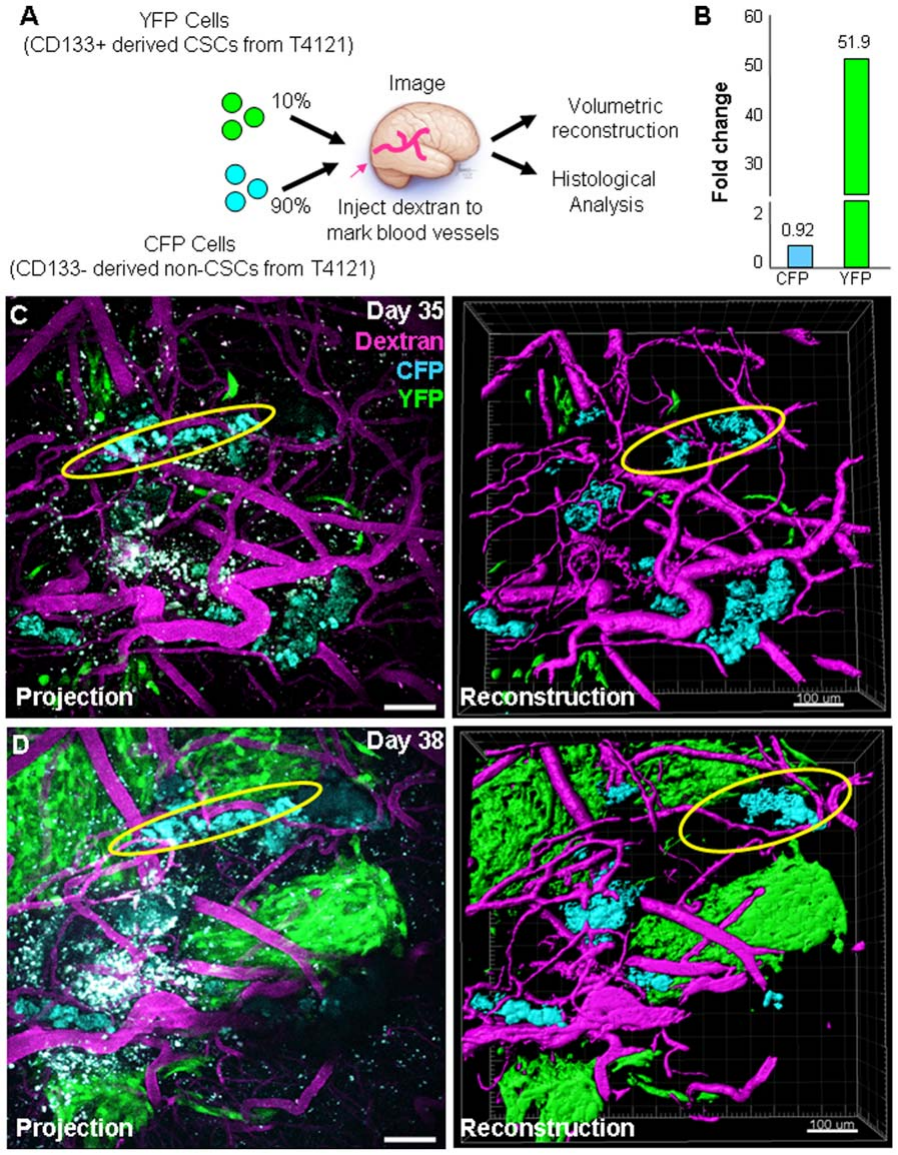
Multiphoton microscopy reveals cancer stem cell driven tumor propagation. Fractionated CSCs and non-stem tumor cells were labeled with different fluorescent proteins and transplanted into mice at a 10% cancer stem cell (YFP) to 90% non-stem tumor cell (CFP) ratio as shown in experimental design schematic (**A**). CSCs outgrew non-CSCs in vivo as shown in summary graph (**B**), which was calculated based on three-dimensional reconstructions of projection micrographs (**B, C**). Additionally, tumor populations did not intermingle in vivo (non-stem tumor population indicated by yellow oval). Fluorescent dextran (shown in purple) was injected into the circulation to illuminate blood vessels prior to imaging. Scale bar represents 100 µm.

Numerous studies have demonstrated differential tumor formation capacity between CSCs and non-stem tumor cells [8], [15], [17], [18], however a head-to-head comparison between the two populations in high resolution or in a time dependent manner has not been performed. This evaluation is critical as a malignant glioma is made up of multiple cell populations, there is cross-talk between the populations, and how the cell populations interact is likely to be informative with regards to tumorigenic processes. To evaluate the behavior of CSCs and non-stem tumor cells in an identical microenvironment, we transplanted differentially labeled human CSCs and non-stem tumor cells derived from the same parental tumor into the same recipient mouse and monitored in vivo behavior over time using intravital microscopy (Fig. 2A). Sequential in vivo assessment of the same host bearing an initial mixture of CSC (10%, YFP labeled) and non-stem tumor cells (90%, CFP labeled) demonstrated that CSCs outgrew non-stem tumor cells, with a 51.9 fold volume increase for the CSCs and a 0.92 fold increase for the non-stem tumor cells (Fig. 2B) and there was limited intermingled growth among populations (Fig. 2C, D). Using multiple cell populations from the same tumor, these imaging results provide the first evidence for differential tumor formation capacity between CSCs and non-stem tumor cells in vivo using high resolution sequential imaging.

### Resulting tumors contained cancer stem cells and their descendants

**Figure 3:**
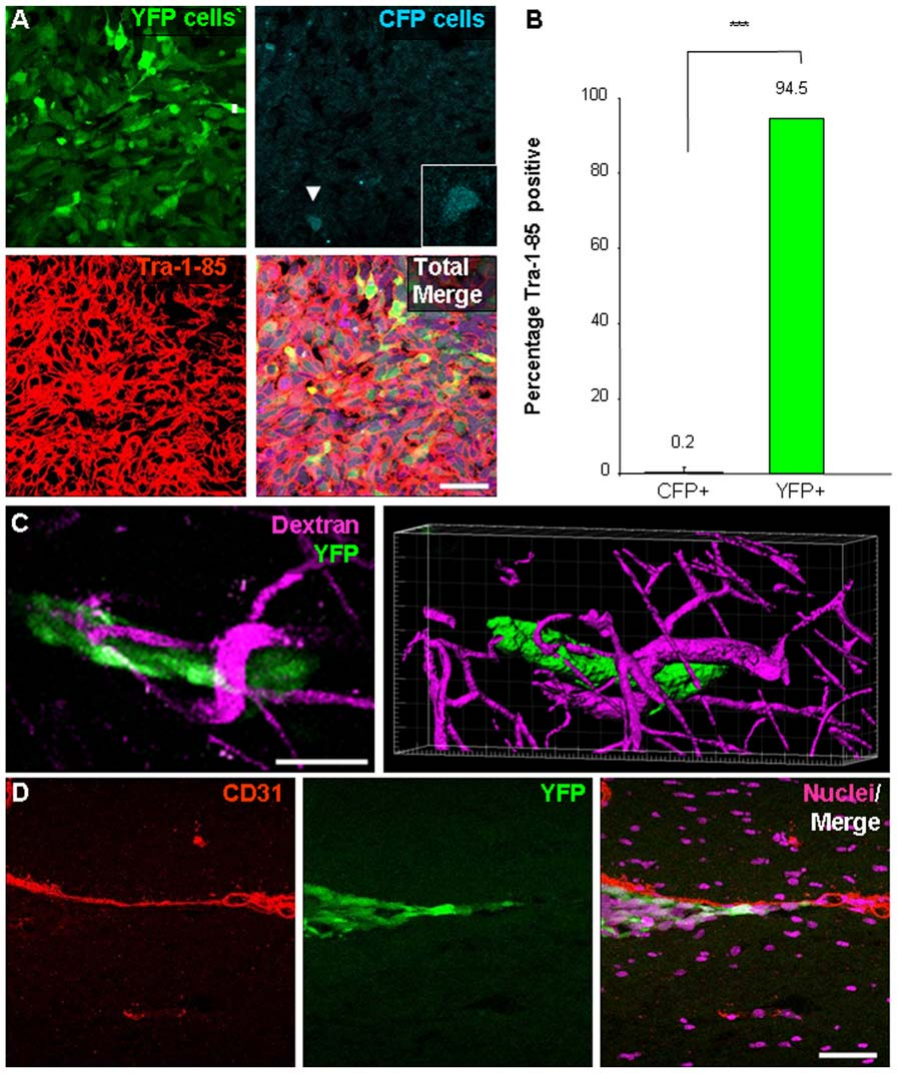
Histological evaluation reveals tumors contained cancer stem cells and their descendants that had association with blood vessels. Tumors from the cell mixing experiments (n = 3) were evaluated to determine their composition. Subsequent evaluation of resulting tumors demonstrates that the majority of the cells within the tumor mass was of human origin and derived from CSC as confirmed by Tra-1-85 staining and YFP expression, shown in representative micrographs (**A**) and bar graph (**B**). Peripheral transplanted tumor cells (YFP positive CSCs and their descendants) were observed to have an association with blood vessels. Micrograph from multiphoton imaging and three-dimensional reconstruction (**C**) depict close association of tumor cells (green) with adjacent blood vessel (purple, illuminated by fluorescent dextran injection into the circulation prior to imaging). Histological examination of resulting tumors confirms close association of peripheral tumor cells to the vasculature using CD31 immunostaining (**D**; CD31 in red, tumor cells in green, nuclei in purple). Scale bar represents 50 µm. Data displayed as mean values +/- S.E.M. ***, p<0.001 as assessed by one-way analysis of variance (ANOVA).

The secondary tumors formed by transplanted CSCs contain multiple cell populations, however previous studies have largely focused on the histology of secondary tumors or expression of tumor markers but there has been limited information with regard to the degree of heterogeneity as well as the contribution of multiple cell types to the development of heterogeneity. After the mice developed neurological signs (ranging from 37 to 42 days after transplantation), we used histological examination to confirm the phenotype of the transplanted cells in the resulting tumors. To delineate the boundaries of the tumors, tissues were stained for a human specific antigen, Tra-1-85. We found that while both YFP positive (CSC derived) and CFP positive (non-stem derived) were detectable, the overwhelming majority of tumor cells were derived from CSCs; 94.5 percent of Tra-1-85 positive cells within the tumor were YFP positive (CSC derived) versus 0.2 percent which were CFP positive (non-stem derived, Fig. 3A, B).

### Association between blood vessels and cancer stem cells and their descendants

The ability for tumor cells to grow along blood vessels is a phenotype that is frequently identified in human glioma surgical specimens. To evaluate if this phenotype was recapitulated in tumors resulting from co-transplantation of CSCs and matched non-stem tumor cells, we assessed tumor vasculature using multiphoton microscopy and histological examination of resulting tumors (Fig. 3C, D). At day 38 (shown in Fig. 2B), were we able to identify a group of tumor cells at the periphery of the tumor and three-dimensional analysis demonstrated that the CSCs and their descendants (YFP cells) were in close proximity to the vasculature (Fig. 3C). Histological examination of resulting tumor using an antibody against CD31 to mark blood vessels also confirmed that CSCs and their descendants (YFP+ cells) were indeed close to the vasculature in regions separated from the bulk tumor mass, a phenomenon also observed in GBM patients (Fig. 3D).

### Cancer stem cells propagate heterogeneity *in vivo*

**Figure 4:**
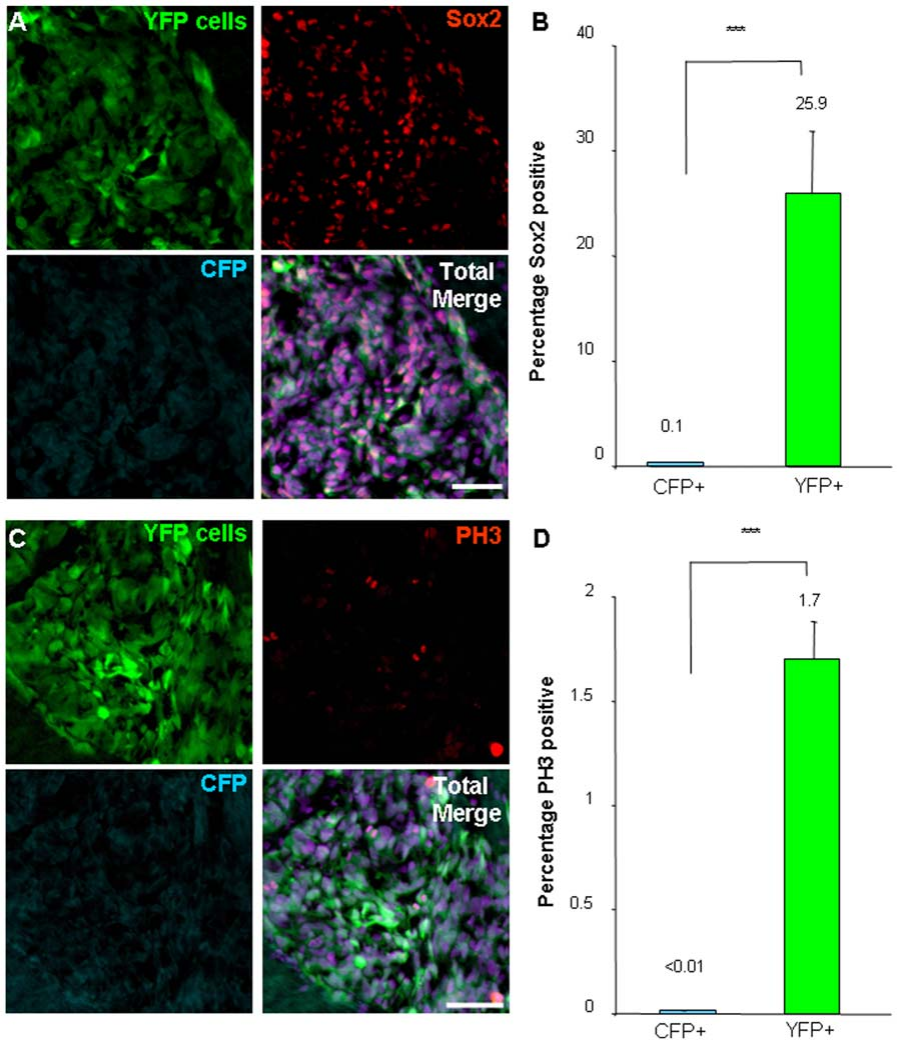
Tumors contain fractions of stem-like and proliferating cells that originated from cancer stem cells. Histological examination was performed from resulting tumors in the cell mixing experiments (n = 3) to determine the fraction of stem-like cells as assessed by Sox2 expression and the presence of proliferating cells as confirmed by the M-phase marker phosphorylated histone 3 (PH3). Representative micrographs (**A**) and bar graph (**B**) demonstrate Sox2 expression (red) is associated with cancer stem cells and their descendants (green) but not with non-stem tumor cells and their descendants (blue). Representative micrographs (**C**) and bar graph (**D**) demonstrate PH3 expression (red) is associated with cancer stem cells and their descendants (green) but not with non-stem tumor cells and their descendants (blue). Scale bar represents 50 µm. Data displayed as mean values +/− S.E.M. ***, p<0.001 as assessed by one-way analysis of variance (ANOVA), nuclei counterstained with Draq5 (purple).

To determine if the transplanted tumor cells contained stem-like cells, we evaluated the expression of the CSC marker, Sox2 [19], and found that 25.9 percent of transplanted CSCs and their descendants (YFP cells) were Sox2 positive as compared to 0.1 percent of non-stem tumor cells and their descendants (CFP cells, Fig. 4A, B). We also assessed proliferation using the M-phase marker phosphorylated-histone H3 (PH3) and found that 1.7 percent of CSCs and their descendants (YFP cells) were actively in M-phase as compared with less than 0.01 percent of non-stem tumor cells and their descendants (CFP cells, Fig. 4C, D). These differences in proliferation seen in our model support clinical reports that suggest proliferating CSCs (based on CD133-positive status) characterize an aggressive class of GBM tumors [20].

**Figure 5:**
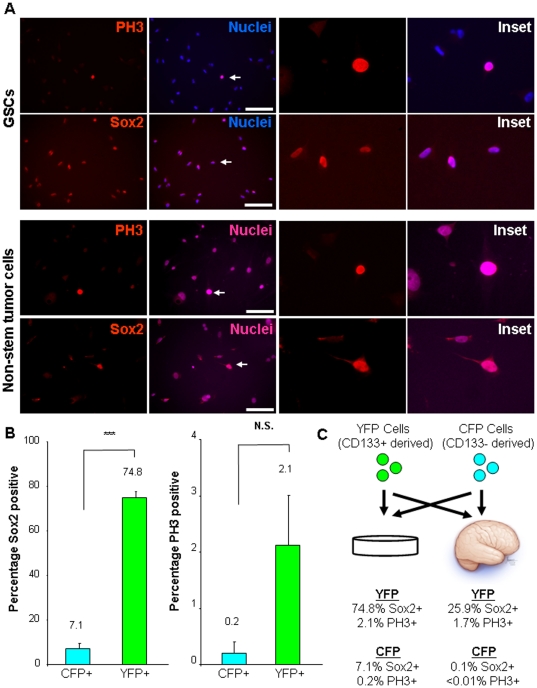
CSCs and non-stem tumor cells prior to transplantation contain different fractions of stem-like and proliferating cells. Representative micrographs (**A**) and bar graph (**B**) of expanded cells prior to transplantation demonstrate Sox2 and PH3 expression (red) is higher in the CSC fraction of cells as compared with the non-stem tumor cells. Summary figure depicts marker expression from in vivo and in vitro analyses (**C**). Scale bar represents 50 µm. Data displayed as mean values +/− S.E.M. ***, p<0.001 and N.S. represents not significant (p>0.05) as assessed by one-way analysis of variance (ANOVA), nuclei counterstained with Hoechst 33342 (blue).

To gain an appreciation for the degree of heterogeneity established by the transplanted CSCs (YFP positive cells), we evaluated the phenotype of the cells prior to transplantation. The CSC cultures contained 74.8 percent Sox2 positive cells (i.e. in vitro) as compared to 24.9 percent of Sox2 positive YFP cells (CSC derived) within the tumor (i.e. in vivo, Fig. 5A–C). These results suggest that the in vivo environment provides instructive cues to recreate an equilibrium of differentiation status and thus cellular heterogeneity. A similar reduction was seen in Sox2 positive non-stem tumor cells (CFP cells) from an initial population of 7.1 percent in culture to 0.1 percent in the tumor (Fig. 5A-C). Differences in growth were also observed between populations in culture as compared to within the tumor. Using the M-phase marker PH3 as a surrogate of proliferation, we observed 2.1 percent YFP positive cells (CSC derived) and 0.2 percent CFP positive cells (non-stem derived) in culture as compared with 1.7 percent YFP positive cells and less than 0.01 percent CFP positive cells in the tumors (Fig 5A–C). These results demonstrate that although non-stem tumor cells predominated at early time points due to the ratios at the time of transplantation, growing tumors had limited numbers of cells derived from non-stem tumor cells, of which few expressed stem cell markers or were actively proliferating. The resulting tumors were comprised of CSCs and their descendants, a fraction of which still contained stem cell marker expression and were actively proliferating.

## Discussion

The tumor microenvironment is a critical regulator of tumor formation and maintenance with contributions to therapeutic response and failure. However, many CSC and brain tumor studies have not appropriately modeled the microenvironment. Often CSC and non-stem tumor cells are grown in different conditions that include different media and CSCs grown as spheres and non-stem tumor cells grown adherently. As these conditions activate different cell signaling pathways, some CSC results may be due to culture artifact rather than intrinsic cellular differences. Many studies are performed with the populations in isolation, which does not allow for signaling between cells that is critical for cell growth or the evaluation of each cell type's contribution to tumor formation in vivo. Furthermore, niche interactions with components such as the vasculature or stroma cannot be fully evaluated unless studies are performed in vivo. To better model the tumor microenvironment, we differentially labeled CSCs and non-stem tumor cells and introduced the cells into the same in vivo conditions. Our evaluation of growth provides the first direct evidence for tumor propagation by a solid tumor CSC subpopulation in vivo using live imaging and shows that a small fraction of tumor cells can propagate a heterogeneous tumor. Our studies offer substantial advantages over ex vivo or matched population studies due to our ability to more appropriate model the in vivo environment and assess the behavior of multiple populations in real time using high resolution microscopy.

There are many aspects of xenotransplantation models that provide potential advantages, however there are still limitations. Conceptually, these transplant assays offer a better in vivo environment at expense of an appreciation for tumorigenic processes in real time, which can only be inferred after tumor formation. However, this black box approach can be addressed with the use of live imaging as described in this report. Using live imaging, the intricacies of the xenotransplantation model can be elucidated and informative predictions can be made for tumor development, maintenance, and response to therapy. Careful use of xenotransplantation models will allow for a greater understanding of the interaction between CSCs and non-stem tumor cells, as well as cellular communication with blood vessels, a CSC niche in several brain tumors [16]. Additionally, these approaches may clarify the recently identified phenomenon of GBM CSC differentiation into vascular cells and clarify the role of this plasticity in tumorigenic processes [21], [22], [23], [24]. Of note, we did not observe integration of labeled cells into the vascular wall (data not shown). These vascular interactions have far reaching therapeutic implications especially in the context of angiogenesis where many therapies are under investigation. In addition, a greater appreciation of the interplay between the microenvironment and transplanted tumor cells may help explain the delay in tumor growth in transplantation models. This is highlighted by our observations shown in Figure 1 where CSC growth between day 35 to day 38 is quite remarkable, but congruent with the dynamics of tumor growth within a transplantation model [25], [26]. These differences are likely to be a reflection of a multi-stage process which includes tumor cell survival, adaptation to the host environment, followed by exponential growth, which can be extrapolated to understand recurrence in patients.

Our data also demonstrate that such an approach can be used to better define cancer cellular heterogeneity, which will be critical for both the basic understanding of tumorigenesis and a model to more appropriately test promising pre-clinical therapies. In our studies, the resulting tumors that formed contained a heterogeneous population, as assessed by Sox2 expression, despite the substantial representation of YFP cells (CSC derived). This type of in vivo lineage tracing has been limited in GBM studies and our results confirm that CSCs expressing a stem cell marker can generate cells that do not express the stem cell marker, demonstrating the transition between stem cell states in vivo. Pre-clinical studies rely on tumor size as an estimation of efficacy. Our demonstration that a small fraction of cancer cells propagates a tumor in vivo represents a complementary approach to evaluate the efficacy of a given treatment by lineage tracing. Taken into a clinical context, a therapy that kills the majority of cells and leaves behind a small population of refractory cells (likely to be enriched in CSCs) will appear effective if tumor size is the measured outcome. However, the remaining cells, some of which are CSCs, are likely to contribute to tumor recurrence, as often seen in patients after therapies effective in reducing tumor size [27]. Hence, evaluating cellular lineages in a tumor after therapy in combination with tumor size assessments and histological analysis is likely to provide a more accurate view of the impact of the treatment. The ability to evaluate tumor cell behavior itself in a relevant clinical model is valuable. Our data (this report and [11], [28]) and those from other groups [16] indicate that tumor cells have an intimate relationship with the vasculature; however, the FDA approved vascular endothelial cell growth factor (VEGF) neutralizing antibody bevacizumab (Avastin) has had mixed success clinically, despite success in pre-clinical models [29]. Resistance to anti-VEGF therapies is postulated to occur through evasion or indifference and involve modulated angiogenesis, vasculogenesis, vascular normalization [30], [31]. However, the dominant mode of resistance is yet to be determined and our approach using multiple tumor cell populations and high resolution in vivo microscopy is likely to provide critical insight. For greater impact, these methods can be combined with use of fluorescent reporter systems to evaluate the transcriptional activity in real time and in correlation to therapeutic response. Another area in which these imaging approaches will be helpful is the evaluation of sonic hedgehog (Shh) inhibitors. It is unclear if the mode of action is directly on tumor cells, the tumor stroma, or both, and elucidation of the mechanism of action is an immediate priority as several Shh inhibitors are currently under investigation for a variety of tumors including GBM [32], [33]. Using the approach described in this report, the examination of CSCs in a context that closely resembles the native microenvironment combined with live imaging will help accelerate our understanding of CSCs and put their associated changes in response to therapy into a relevant context.
